# Single Cell Analysis of Endothelial Cells Identified Organ-Specific Molecular Signatures and Heart-Specific Cell Populations and Molecular Features

**DOI:** 10.3389/fcvm.2019.00165

**Published:** 2019-11-26

**Authors:** Wei Feng, Lyuqin Chen, Patricia K. Nguyen, Sean M. Wu, Guang Li

**Affiliations:** ^1^Department of Developmental Biology, University of Pittsburgh School of Medicine, Pittsburgh, PA, United States; ^2^Department of Neurosurgery, Stanford University School of Medicine, Stanford, CA, United States; ^3^Department of Pharmacology and Chemical Biology, UPMC Hillman Cancer Center, Magee-Womens Research Institute, University of Pittsburgh, Pittsburgh, PA, United States; ^4^Cardiovascular Institute, Stanford University School of Medicine, Stanford, CA, United States; ^5^Veterans Affairs Palo Alto Health Care Administration, Palo Alto, CA, United States; ^6^Division of Cardiovascular Medicine, Department of Medicine, Stanford University School of Medicine, Stanford, CA, United States; ^7^Institute for Stem Cell Biology and Regenerative Medicine, Stanford University School of Medicine, Stanford, CA, United States

**Keywords:** endothelial cell, single cell mRNA sequencing, lymphatic vascular, germ layer, heart, aorta

## Abstract

*Endothelial cells line the inner surface of vasculature and play an important role in normal physiology and disease progression*. Although most tissue is known to have a heterogeneous population of endothelial cells, transcriptional differences in organ specific endothelial cells have not been systematically analyzed at the single cell level. The Tabula Muris project profiled mouse single cells from 20 organs. We found 10 of the organs profiled by this Consortium have endothelial cells. Unsupervised analysis of these endothelial cells revealed that they were mainly grouped by organs, and organ-specific cells were further partially correlated by germ layers. Unexpectedly, we found all lymphatic endothelial cells grouped together regardless of their resident organs. To further understand the cellular heterogeneity in organ-specific endothelial cells, we used the heart as an example. As a pump of the circulation system, the heart has multiple types of endothelial cells. Detailed analysis of these cells identified an endocardial endothelial cell population, a coronary vascular endothelial cell population, and an aorta-specific cell population. Through integrated analysis of the single cell data from another two studies analyzing the aorta, we identified conserved cell populations and molecular markers across the datasets. In summary, by reanalyzing the existing endothelial cell single-cell data, we identified organ-specific molecular signatures and heart-specific subpopulations and molecular markers. We expect these findings will pave the way for a deeper understanding of vascular biology and endothelial cell-related diseases.

## Introduction

As a universal cell type, endothelial cells exist in the whole body, lining the inner surface of vasculature to build a barrier between blood and tissue cells. The cells in each organ have been known to be heterogeneous, which includes structural differences such as cell size, cell morphology and intercellular junctions, and functional differences such as cell permeability, cell homeostasis, leukocyte trafficking, angiogenesis, and innate and acquired immunity (1–4). These heterogeneities have been extensively characterized on the cellular and molecular level, but the molecular analysis were largely carried out on the level of cell populations (1, 4, 5). Because of there may be subtle differences that dictate endothelial cell heterogeneity, understanding the molecular differences at single-cell level is required.

Single-cell mRNA sequencing as an advanced tool has achieved significant progress in the last several years. It has been widely used to characterize the cellular heterogeneity in different tissues and to identify molecular markers and rare cell populations (6). Currently, SMART-seq2 based methods and 10x genomics solutions are the two most popular approaches (7). The SMART-seq2 is able to profile the full length of transcripts but has a relatively low throughput, and 10x Genomics solutions can easily capture a large number of single cells but can only profile the 5′ or 3′ portion of transcripts (7, 8). Besides single-cell mRNA sequencing, single molecular *in situ* hybridizations also have made important progress. Multiple methods have been developed to analyze the genes expression in single cells *in situ*. Based on rolling cycle amplification (RCA), Proximity Ligation *in situ* Hybridization (PLISH) is one of the most cost-effective strategies and has been used to analyze genes expression in multiple tissues (9).

With SMART-Seq2 and 10x Genomics solutions, the *Tabula Muris* project profiled ~100,000 single cells in 20 organs which were derived from four male and three female mice (10). The heart as one of the 20 organs was profiled with both approaches. About 4,000 single cells were profiled with SMART-Seq2, and each heart was micro-dissected into five zones including left atrial (LA), right atrial (RA), left ventricular (LV), right ventricular (RV), and aorta. Around 650 single cells were profiled with the 10x Genomics solution and no anatomical segregations were made in this procedure (10).

The heart is known to have multiple types of endothelial cells. Not only are the heart chambers lined with a layer of endocardial endothelial cells to separate the cardiac muscle tissue from the circulating blood, the myocardial tissue in the heart chambers also surrounds a network of coronary vasculature, which mainly function to supply oxygen to the myocardial cells. The coronary vascular endothelial cells can be further separated into artery, venous, and capillary cells based on their morphological and functional differences. In addition, large vessels such as aorta also have multiple types of endothelial cells (11). Besides blood vessels, lymphatic vessels also exist in heart and connect to the lymph nodes in the whole body to rid waste and transport lymph (12).

To analyze the cellular and molecular heterogeneity of endothelial cells in heart and other organs, we have made a detailed analysis of the endothelial cell single cell transcriptional profile from the *Tabula Muris* project. We also have integrated the *Tabula Muris* data with other single cell data sets to identify conserved cell populations and molecular markers in different studies.

## Results

### Identification of Molecular Signatures in Organ-Specific Endothelial Cells

**Figure 1:**
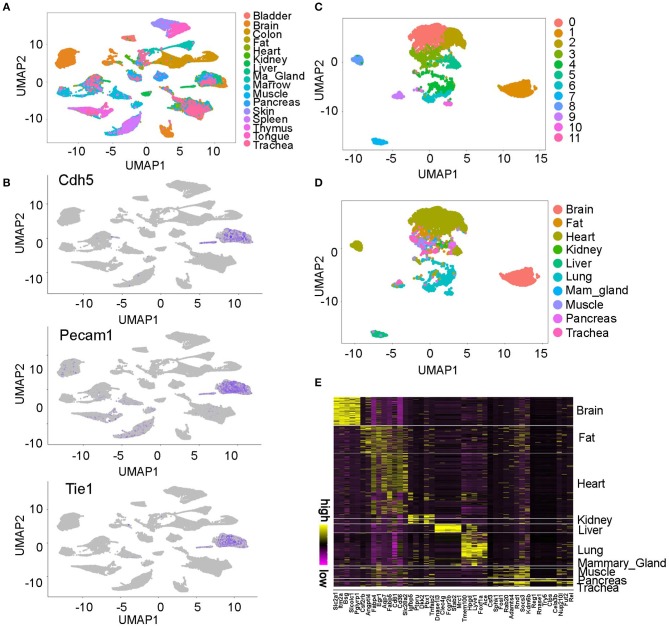
Overview of the endothelial cell populations profiled in *Tabula Muris* project. **(A)** UMAP plot of the single cells profiled in *Tabula Muris* project. This figure is based on Figure 2 in Tabula Muris study (10), reproduced under the CC-BY license. **(B)** Two clusters were consistently labeled by the EC marker gene Cdh5, Pecam1, and Tie1. **(C,D)** ECs were grouped into 12 clusters and most cells were grouped by organ. **(E)** The molecular signatures in each organ-specific ECs are shown. The top five genes in each organ were plotted on the heatmap. All the figures were generated using the Tabula Muris data (10).

The *Tabula Muris* project profiled single cells from 20 adult-staged mouse organs (10). The unsupervised analysis found most cells were grouped by organs, but some clusters were contributed by cells from multiple organs (Figure 1A). To identify the endothelial cells in the data, we analyzed the expression pattern of endothelial cell lineage gene Pecam1, Cdh5, and Tie1 (Figure 1B) (13). We found two cell clusters highly express all three genes and we annotated them as *endothelial cells (ECs)*. Further analysis of these ECs revealed they were derived from 10 organs and grouped into 12 clusters on UMAP plot (Figures 1C,D). *Interestingly, we found a gender difference between male and female cells in some organs such as lung but not in others such as brain* (Figure S1A). Furthermore, we identified genes that specifically express in each organ derived ECs through differential gene expression analysis. For example, we found the brain ECs uniquely express Slc2a1 and Itm2a, and the liver ECs specifically express Dnase113 and Clec4g (Figure 1E, Table S1).

### Identification of a Cluster of Lymphatic Endothelial Cells Contributed by Multiple Organs

**Figure 2:**
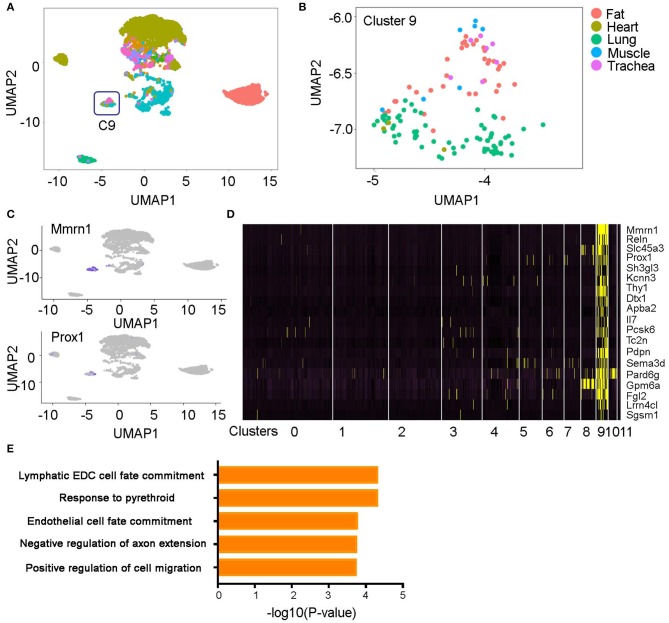
A lymphatic endothelial cell population (e.g., Cluster 9). **(A,B)** The ECs in cluster 9 originated from multiple organs. **(C,D)** Unique genes in cluster 9 were identified and shown on the UMAP plots and heatmap. **(E)** Pathway analysis of the cluster 9 specific genes is shown. All the figures were generated using the Tabula Muris data (10).

Within the 12 EC clusters, most were sourced from a single organ (Figures 1C,D, 2A). Exceptionally, cluster 9 as an independent cluster consists of cells from multiple organs including fat, heart, muscle, lung, and trachea (Figure 2B). Through differential gene expression analysis, we found a list of genes including Mmrn1, Prox1, and Pdpn that specifically express in this cluster (Figures 2C,D, Table S2). Prox1 and Pdpn were reported to express in lymphatic ECs to regulate cell lineage development (14, 15), and gene pathway analysis of the cluster-specific genes revealed a significant enrichment of lymphatic cell fate commitment and positive regulation of cell migration pathways (Figure 2E). These results suggest cluster 9 is a lymphatic EC population existing in multiple organs.

### Correlation Analysis of ECs From Different Organs

**Figure 3:**
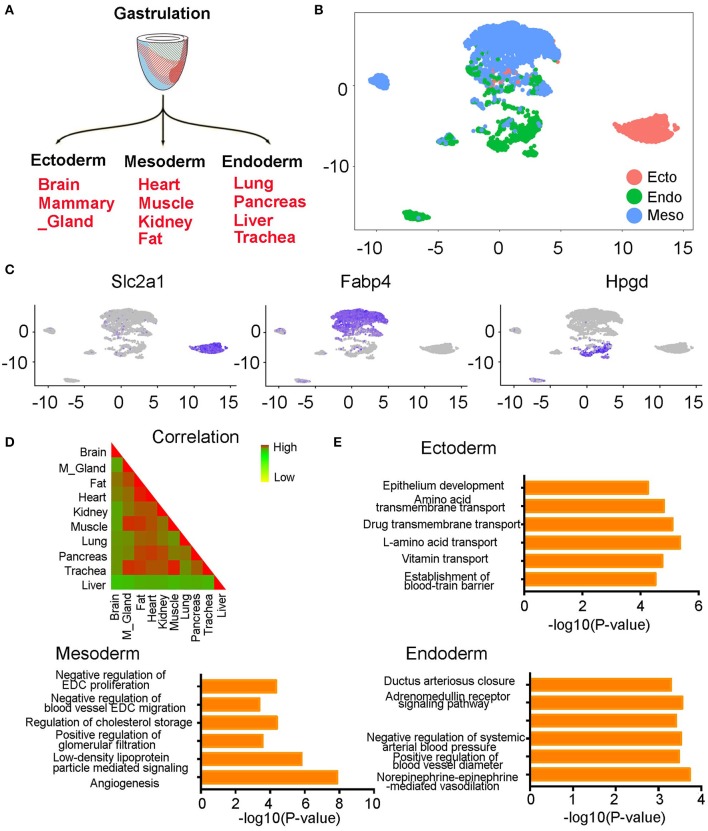
The endothelial cells from different organs partially grouped by germ layers. **(A)** Diagram showing the development of each germ layer into different organs. **(B)** The ECs were partially grouped by germ layers on UMAP plot. **(C)** The expression pattern of germ layer-specific genes is shown. **(D)** The Pearson correlation coefficients of ECs at different organs is shown. **(E)** Bar chart showing gene pathway analysis of the genes specifically expressed in each germ layer derived ECs. **(B–E)** were generated using the Tabula Muris data (10).

At early developmental stages, the single-layered blastula develops into a three-layered structure through a process called gastrulation. The three germ layers are known as ectoderm, mesoderm, and endoderm. Each germ layer will further develop into multiple tissues. The ectoderm develops into the brain and mammary gland, mesoderm develops into heart, muscle, kidney, and fat, and endoderm develops into lung, pancreas, liver, and trachea (Figure 3A) (16). When we annotated the ECs with their germ layer information, we observed a general cell separation by germ layers on the UMAP plot. The ectoderm cells mainly grouped on the right side of the plot, mesoderm cells on the top part of the plot, and endoderm cells in the middle and bottom part of the plot (Figure 3B). When we further calculated the Pearson correlation coefficients between each organ-derived ECs, we found a consistent result. Specifically, we found that the brain derived from an ectodermal origin and liver derived from an endoderm origin have the lowest coefficients with the other tissues. On the other hand, the mesodermal fat, heart, kidney, and muscle have very high coefficients with the other mesodermal tissues. Finally, the lung, pancreas, and trachea originating from the endoderm also have high coefficients with each other (Figure 3D). Specific genes in each germ layer derived cells were further identified (Table S3). For example, we found that Slc2a1, Fab4, and Hpgd are highly expressed in ectoderm, mesoderm, and endoderm-derived cells, respectively (Figure 3C). Pathway analysis of these genes found the ectoderm ECs enrich in amino acid and vitamin transport pathways, mesoderm ECs are mainly involved in active angiogenesis, low-density lipoprotein particle-mediated signaling, and regulation of EC proliferation and migration pathways, and endoderm ECs highly express genes in norepinephrine-epinephrine mediated vasodilation, regulation of blood vessel diameter, and ductus arteriosus closure pathways (Figure 3E).

### The Endothelial Cell Populations in Cardiac Chambers

**Figure 4:**
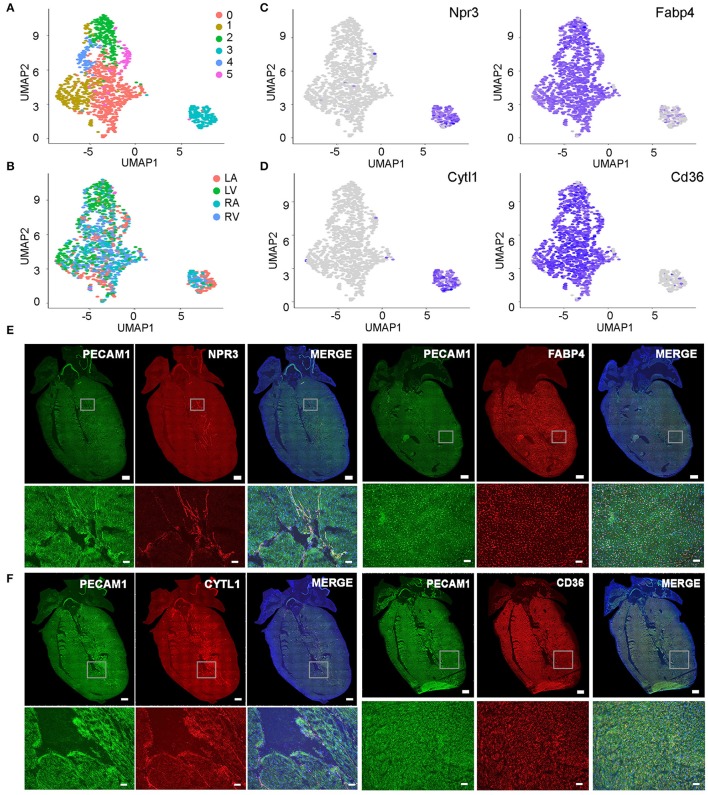
Subpopulation analysis of cardiac endothelial cells. **(A,B)** Cardiac ECs were grouped into six clusters on UMAP plot but no obvious chamber-specific pattern was observed. LA, left atrial; RA, right atrial; LV, left ventricular; RV, right ventricular. **(C)** The expression pattern of endocardial EC gene Npr3 and coronary vascular EC gene Fabp4 is shown. **(D)** The UMAP plots showing the expression of Cytl1 and Cd36. **(E,F)** Analysis of gene expression pattern by single molecular *in situ* hybridization. Note endocardial ECs specifically express Npr3 and Cytl1, and coronary vascular ECs specifically express Fabp4 and Cd36. Scale bar in the whole heart sections are 500 μm and in the enlarged images are 50 μm. **(A–D)** were generated using the Tabula Muris data (10).

Considering each organ has multiple types of ECs, we used the heart as an example to analyze the organ-specific subpopulations. As the engine of the circulatory system, the heart has a very complicated vascular system. Unsupervised analysis of cardiac ECs identified two cell populations and six cell clusters (Figure 4A). No obvious chamber-specific pattern *and gender differences were observed in these cells* (Figure 4B, Figure S1B). Through gene expression analysis, one cell population was found to highly express Npr3 and the other expresses Fabp4 (Figure 4C). Npr3 and Fabp4 were reported to, respectively, express in endocardial ECs and coronary vascular ECs at embryonic stages (17). *In situ* hybridization confirmed that they also specifically labeled the two cell populations at adult stages (Figure 4E, Figure S3). Furthermore, we identified a novel endocardial endothelial cell gene Cytl1 and confirmed the expression of Cd36 as a coronary vascular endothelial cell gene. In addition, we further validated their expression pattern with *in situ* hybridizations (Figures 4D,F, Figure S3).

### Stage-Specific Molecular Features in Endocardial Endothelial Cells

**Figure 5:**
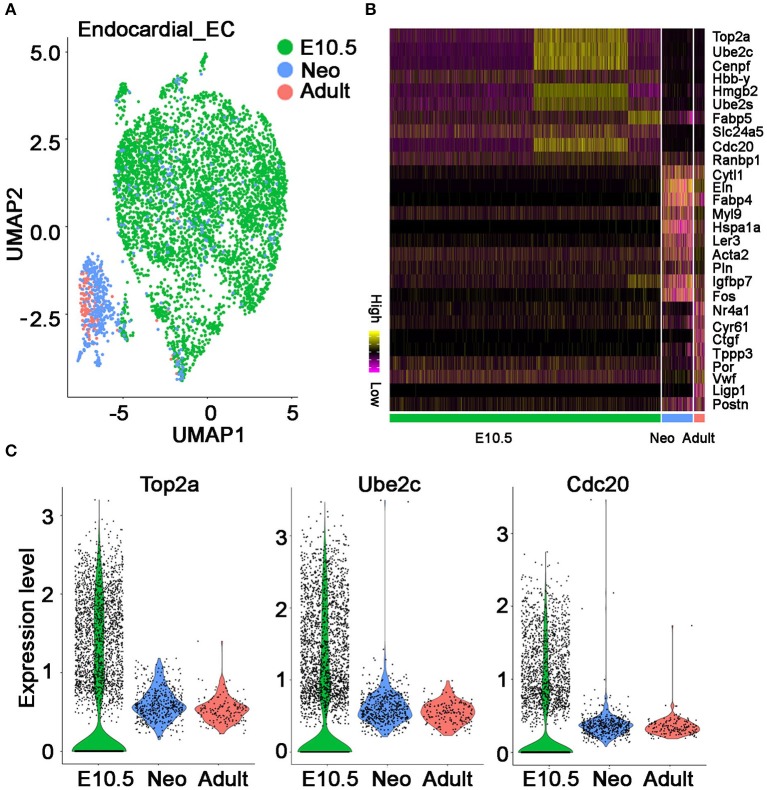
The molecular features of endocardial endothelial cells at different developmental stages. **(A)** The UMAP plot showing endocardial ECs at three developmental stages. **(B)** The top 10 genes specifically expressed at each stage are shown. **(C)** Violin plot showing cell cycle gene Top2a, Ube2c, and Cdc20 at each stage. **(A–C)** Were generated using the Tabula Muris data (10) and Microwell mouse cell atlas data (18).

The development of endocardial ECs starts at the early embryonic stages. Through integrated analysis of the endocardial ECs at embryonic day 10.5 stage, neonatal P1 stage and adult stage (18, 19), we found on UMAP plot all the embryonic cells have a similar transcriptional profile and group together; whereas, neonatal and adult cells are more similar and group into another cluster (Figure 5A). The stage-specific genes were further identified by differential analysis (Figure 5B). Interestingly, we found the cell cycle genes such as Top2a, Ube2c, and Cdc20 are highly expressed at embryonic cells but significantly reduced at neonatal and adult cells (Figure 5C), suggesting the endocardial ECs dramatically decreased their proliferation activities starting from neonatal P1 or even early stages. *This observation is consistent with a previous finding made with a cell cycle fluorescence reporter in heart and other organs, in which they found a decline in EC proliferation between E9.5 to E12.5, and most ECs are quiescent at adult stages* (20).

### Identification of an Aorta-Specific Endothelial Cell Population

**Figure 6:**
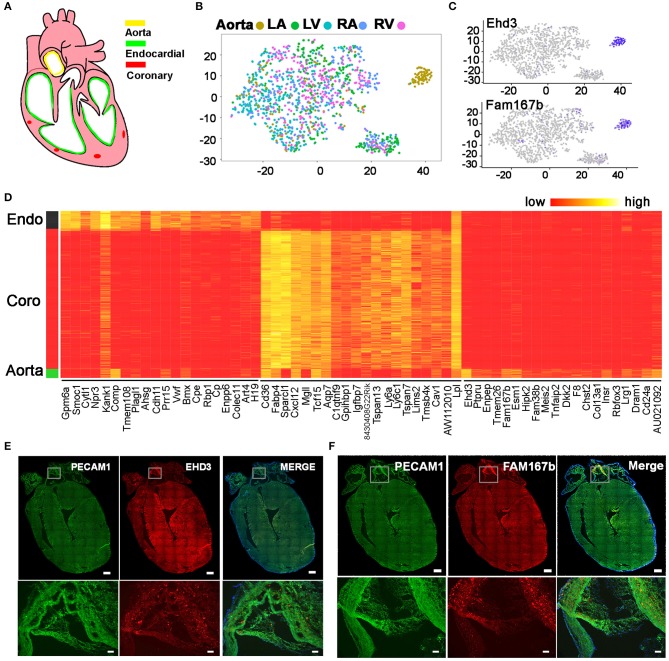
Identification of an aorta-specific endothelial cell population. **(A)** Diagram showing the heart endothelial cell distribution. **(B)** Unsupervised analysis of ECs from aorta and heart chambers was performed. **(C)** Aorta-specific EC population specifically express Ehd3 and Fam167b. **(D)** The top 20 genes that are specifically expressed in endocardial ECs, coronary vascular ECs, and aorta-specific ECs. **(E,F)** The pattern of Ehd3 and Fam167b expression was validated by single molecular *in situ* hybridization. Scale bar in the whole heart sections are 500 μm, and in the enlarged images are 50 μm. **(B–D)** Were generated using the Tabula Muris data (10).

Besides heart chambers, ECs also exist in large cardiac vessels such as aorta (Figure 6A). Combined analysis of the ECs from heart chambers and aorta identified three populations. *Besides the endocardial EC and coronary vascular EC populations, which consist of cells from the heart chambers and aorta*, we also found an aorta-specific cell cluster (Figure 6B). Through differential gene expression analysis, we identified genes that uniquely express in each of the three EC populations (Figure 6D, Table S4). Ehd3 and Fam167b as the top 2 genes that specifically express in the aorta-specific EC cluster were further validated with *in situ* hybridization. The hybridization signals clearly showed both genes highly express in aorta cells but not in heart chamber cells (Figures 6C,E,F, Figure S3).

### Integrated Analysis of Aorta Endothelial Cells From Multiple Studies

**Figure 7:**
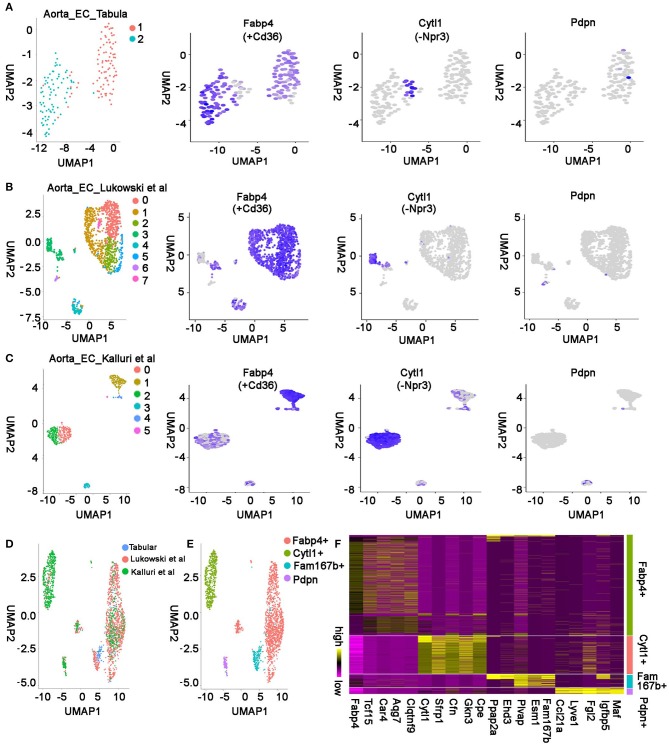
Integrated analysis of aorta endothelial cells in multiple studies. **(A)** Unsupervised analysis of the aorta ECs from *Tabula Muris* study and representative genes in each cell population are shown. **(B)** UMAP plot showing cell clusters and cluster-specific genes in Lukowski et al. study. The first panel in this figure is based on Figure 1B in Lukowski et al. study (22), reproduced under the CC-BY license. **(C)** The single-cell RNA sequencing data in Kalluri et al. study is shown. The first panel in this figure is based on Figure 1D in Kalluri et al. study (21), reproduced under the CC-BY license. **(D)** Unsupervised analysis of the integrated data from all three studies is displayed. **(E)** The integrated data were grouped into four types of ECs. **(F)** Marker genes in each type of EC were identified by differentially expression analysis of the integrated data. Top 5 genes in each population have been presented. All the figures were generated using the data from Tabular muris project (10), Lukowski et al. study (22), and Kalluri et al. study (21).

There were two other aorta studies with single-cell mRNA sequencing recently (21, 22). To determine if the same endothelial cell populations were captured at the three studies, we selected the ECs based on the expression of Pecam1, Cdh5, and Tie1 in each study and used unsupervised analysis to analyze their cell populations. In the *Tabula Muris* study, *170* aorta-derived ECs were identified and grouped into two clusters on UMAP plot. The cluster 1 cells highly express Ehd3 and Fam167b, which are the top two marker genes in aorta-specific ECs; Cluster 2 cells mostly express coronary vascular EC marker gene Fabp4 and Cd36, but a few cells highly express Cytl1 but not Npr3, which are the top two genes expressing in endocardial ECs, suggesting these aorta cells are endocardial EC like cells but express a few genes differently (Figure 7A, Figure S2). In the Lukowski et al. study, *1,160* ECs grouped into 8 clusters. Five clusters were Fabp4+/Cd36+, one cluster was Cytl1+/Npr3–, and one cluster of cells expressed Fam167b but not Ehd3, suggesting the Ehd3 expression in the aorta-specific EC population was variable in different studies (Figure 7B, Figure S2) (22). In the Kalluri et al. study, *750* ECs formed 6 clusters. Two cluster cells were Fabp4+/Cd36+, three clusters were Cytl1+/Npr3–, and one cluster expressed lymphatic gene Pdpn and Fgl2. *However, we did not identify a cell cluster that specifically express Fam167b or its highly correlated genes in this study* (Figure 7C) (21).

Next, to systematically compare the aorta cells in the three studies, we integrated all three datasets together using an “anchor” strategy in Seurat and grouped them further with unsupervised analysis. Consistently, we found the Fabp4+ and Cytl1+ cells were present in all three datasets, the Fam167b+ population was only captured in the *Tabula Muris* study and Lukowski et al. study, and the Pdpn+ population was only found in the Lukowski et al. and Kalluri et al. study (Figures 7D,E). *These results suggest that most cell populations are conserved across the different studies, but some are only captured by one or two methods, resulting from different causes such as a different number of cells profiled in the three studies. In addition, we found the Fam167b*+ *cells in the Tabula Muris study were mainly from male mice* (Figure S1C). *Considering these cells were also identified in the female mice in the Lukowski et al. study, this phenomena is probably due to the small and unequal number of cells profiled between male and female mice in the Tabula Muris study*. To identify the genes that are conserved across the studies, we analyzed the integrated aorta EC data and found the genes that specifically express in each group of cells (Figure 7F, Table S5). We found Fabp4 and Tcf15 specifically expressed in the Fabp4+ coronary EC population, Cytl1 and Sfrp1 highly expressed in the Cytl1+ endocardial like EC population, Fam167b and Esm1 highly expressed in the Fam167b+ aorta-specific EC population, and Ccl21a and Lyve1 highly and specifically expressed genes in the Pdpn+ lymphatic EC population (Figure 7F, Table S5).

## Discussion

Through the analysis of *Tabula Muris* single cell data, we identified ECs in 10 organs and found each organ-specific molecular signatures. We also found all lymphatic ECs grouped together regardless of their resident tissues. In heart ECs, we identified endocardial ECs, coronary vascular ECs, and aorta-specific ECs. Furthermore, through the integrated analysis of aorta cells from three studies, we identified the conserved EC populations and their marker genes.

Correlation analysis of the ECs at different tissues found the brain and liver ECs have the lowest correlation with other tissues, and the ECs in mesoderm and endoderm-derived organs prefer to group together by germ layers (16). Consistently, analysis of the capillary EC microarray profiles also found most mesoderm tissues including kidney, heart, muscle, spleen, and bone marrow are tightly correlated with each other, while the ECs in liver, testis, and brain have the lowest correlations with other tissues (4). Besides germ layers, we think some other factors could also contribute to the tissue correlations. For example, the transcriptional profile of ECs is significantly remodeled by their physiological function and tissue environment at adult stages. The organs with similar function or close anatomical locations may have correlated gene expression profiles. For example, ECs in the heart appear most similar to the lung ECs, possibly due to their adjacent anatomical position*. In addition, each organ has multiple types of ECs, such as the ECs in artery, vein, and capillaries, which are known to have different functions, cell shapes, and molecular markers. The anatomical location and cell percentage of these ECs are also highly adapted to the underlying tissues, and those differences have been observed between the vascular beds in heart, lung, kidney, and liver* (5). The correlation level between organs will be significantly affected by the actual percentage of each EC type and the percentage that were successfully captured by the single cell approaches.

Within heart chambers, two EC populations were identified. One is coronary vascular ECs and the other is endocardial ECs. These two cell types started to develop at early developmental stages (17). It is interesting to see how their transcriptional profiles have changed along the developmental process. In this study, we made an integrated analysis of endocardial ECs at three developmental stages, and we found neonatal and adult staged cells are different from the cells at the embryonic stage by dramatically reducing their cell cycle genes. For the coronary vascular ECs, we previously compared the E12.5 staged cells with adult staged cells, we found the embryonic artery cells were most similar to the adult arterial cells, and embryonic coronary vessel plexus cells were most similar to adult venous and capillary cells, suggesting each cell type at embryonic stage and adult stage were generally matched (23). However, we also identified genes including Notch1 that differentially expressed in artery populations at these two stages, suggesting the transcriptional profiles of coronary vascular ECs were also significantly remodeled from embryonic stages to adult stages.

Through an integrated analysis of aorta ECs from three studies, we found different cell populations and molecular marker genes in each dataset. These differences could be caused by different reasons. The aorta data from *Tabula Muris* project was profiled with a FACS based SMART-seq2 method, and the other two datasets were generated with 10x Genomics solutions. Each of the two methods has its own cell size preferences and could capture different cell populations. In addition, each method relies on their own specific reagents which could have different sensitivity in detecting genes expression. Furthermore, the mouse age could also have an impact on the cell population and genes expression although C57BL/6 mice have been used in all three studies. The mouse age in Lukowski et al. study was not specified, and the *Tabula Muris* and Kalluri et al. (21) used 3 months and 12 weeks old mice, respectively. Lastly, the differences in tissue dissection and single cell preparations can also have a direct impact on the result differences.

*Via analysis of publically available single cell RNA sequencing data*, we have identified a set of novel genes in each EC population in this study. Our next step will be to analyze their function in EC lineage development and homeostasis. Transcription factors and epigenetic factors as important lineage regulators can be specifically knocked out in ECs with the genome editing techniques such as CRISPR/Cas9. In addition, it will be interesting to analyze the cell-cell interactions between ECs and other cell types in each organ. For example, because endocardial ECs in heart have been reported to restrict cardiomyocyte proliferation at early developmental stages through non-cell autonomous mechanisms (19), it will be interesting to test if there is an inhibitory role of endocardial ECs on cardiomyocyte division at adult stages.

## Materials and Methods

### Single Molecular *in situ* Hybridization Staining With PLISH

For PLISH staining, 2 male C57BL/6J mice at 3 month age were used. All mice were manipulated by following the protocol approved by the Administrative Panel on Laboratory Animal Care (APLAC) at Stanford University and the University of Pittsburgh.

The PLISH experiments were carried out by following the published protocol with minor modifications (9, 19). Briefly, hearts were collected from 3 months old C57BL/6J mice, and embedded into OCT with cold isopentane, which was chilled with liquid nitrogen and dry ice. Twenty micrometer of sections were then cut from the tissue and stored at −80 freezers. When used, the sections were dried for 10 min at room temperature and post-fixed with 3.7% formaldehyde/0.1% DEPC for 30 min. After briefly rinsing with PBS, the sections were treated with 0.1 mg/ml of pepsin for 20 min at 37 degrees. Furthermore, the sections were sequentially rinsed with PBS/60% ethanol/85% ethanol/100% ethanol and dried at room temperature.

For hybridization, sections were first incubated with H probes (Table S6) in H hybridization buffer (1 M sodium trichloroacetate, 50 mM Tris pH 7.4, 5 mM EDTA, 0.2 mg/mL Heparin) for 2 h, and then moved to bridge-circle buffer (2% BSA, 0.2 mg/mL heparin, 0.05% Tween-20, 1X T4 ligase buffer in RNase-free water) for 1 h and ligation buffer (10 CEU/ml T4 DNA ligase, 2% BSA, 1X T4 ligase buffer, 1% RNaseOUT, and 0.05% Tween-20 in RNase-free water) for 2 h. After that, the sections were kept in RCA buffer (1 U/ml Nxgen phi29 polymerase, 1X Nxgen phi29 polymerase buffer, 2%BSA, 5% glycerol, 10 mM dNTPs, 1% RNaseOUT in RNase-free water) overnight. Finally, the sections were stained with labeling buffer (2x SSC/20% formamide in RNase-free water) for 1 h.

The staining signal was imaged under 10x objective with AxioImage epifluorescence microscope at Stanford Neuroscience Microscope Service (NMS) facility, and the images for the whole heart sections were tile together with multiple images taken under the 10x objective.

### Single-Cell mRNA Sequencing Data

The SMART-seq2 data from *Tabula muris* project was downloaded from figshare (https://figshare.com/projects/Tabula_Muris_Transcriptomic_characterization_of_20_organs_and_tissues_from_Mus_musculus_at_single_cell_resolution/27733) (10). The embryonic day 10.5 endocardial ECs data was downloaded from Gene Expression Omnibus (GSE122403) (19). The neonatal endocardial ECs data was downloaded from figshare (https://figshare.com/articles/MCA_DGE_Data/5435866) (18). The Lukowski et al. and Kalluri et al. aorta single-cell RNA sequencing data were, respectively, downloaded from the Broad Institute Single Cell Portal (https://portals.broadinstitute.org/single_cell/study/SCP289/single-cell-analysis-of-the-normalmouse-aorta-reveals-functionally-distinct-endothelial-cell-populations) and ArrayExpress (E-MTAB-7149) (21, 22).

### Single Cell Data Analysis

The *Tabula muris* data was processed in Seurat V2 as described previously (10) and the Seurat objects were downloaded from the *Tabula muris* figshare page. The expressions of Cdh5, Pecam1, and Tie1 were used to identify the endothelial cells in each tissue. Only the cell populations that express all the three EC genes were selected and merged into a single Seurat object for the downstream analysis in Seurat V3 (24).

All the other single cell data including the neonatal endocardial EC data which were generated with Microwell, and embryonic endocardial EC and aorta EC data which were profiled with 10x Genomics solution, were analyzed in Seurat V3 (24). The analysis pipeline in Seurat V3 is similar to the one in Seurat V2, which has been described previously (19). The main update in Seurat V3 is that it uses selection method “vst” to find variable features and uses all genes to scale the data.

To integrate the endocardial and aorta EC data from different sources, we used an “anchor” strategy in Seurat V3 (24, 25). Specifically, this strategy first reduced the dimensionality of all datasets using diagonalized canonical correlation analysis (CCA) (24), then it applied L2-normalization to the canonical correlation vectors. After that, it searched for mutual nearest neighbors (MNNs) in the shared low-dimensional representation, and the resulting cell pairs were referred to as anchors, as they encode the cellular relationships across datasets. Next, a scoring procedure was used to assign a score to each anchor pair based on the shared overlap of mutual neighborhoods for the two cells in a pair, and the anchors with low-scoring correspondence were filtered. Furthermore, to assemble a reference of the multiple single cell datasets, a process similar to batch correction was applied. To do that, a batch vector representing the difference in expression profiles between the two cells in each anchor was firstly calculated. Then a correction vector representing a weighted average across multiple batch vectors was calculated, and these weights were determined by a cell similarity score and the anchor score. These weighted correction vectors were further subtracted from the query gene expression matrix to generate a corrected query expression matrix which can then integrate with the original reference dataset. To integrate multiple datasets, a guide tree based on the similarity between all pairs of datasets were built and proceeded with recursive pairwise correction up the tree (25).

In Seurat V3, we first used the function FindIntegrationAnchors to identify anchors between the datasets, and then used the function IntegrateData to integrate all three datasets based on the anchors. After that, the integrated data can be treated as a single dataset for the followed analysis. All the ECs at E10.5 stage and only the Npr3+ ECs at neonatal and adult stages were identified as endocardial ECs and used for the integration analysis, and all ECs from the aorta studies were integrated.

### Other Analysis

The gene ontology pathways were calculated on Gene Ontology Consortium website (http://geneontology.org/). Specifically, the top 50 genes that highly express in cluster 9 and top 100 genes that highly express in each germ layer cells were used for gene pathway analysis and generating the Figures 2E, 3E, respectively. In those plots, the *P* values represent the probability of seeing at least X number of genes out of the total N genes in the list annotated to a particular GO term, given the proportion of genes in the whole genome that are annotated to that GO Term (The N represents the total number of genes we have used in the analysis, here is 50 or 100; The X represents the observed genes in each pathway in the 50 or 100 genes). In addition, we calculated the –log_10_ of *P* values and used them to generate the plots.

All histograms were plotted in Prism 7, and Pearson correlation heatmap was plotted in R 3.6.1. The supervised heatmaps in Figures 1E, 2D, 7F were generated using the doheatmap function from Seurat package in R 3.6.1.

## Data Availability Statement

Publicly available datasets were analyzed in this study. This data can be found here: GSE109774, https://figshare.com/projects/Tabula_Muris_Transcriptomic_characterization_of_20_organs_and_tissues_from_Mus_musculus_at_single_cell_resolution/27733, GSE122403, https://figshare.com/articles/MCA_DGE_Data/5435866, https://portals.broadinstitute.org/single_cell/study/SCP289/single-cell-analysis-of-the-normalmouse-aorta-reveals-functionally-distinct-endothelial-cell-populations, E-MTAB-7149.

## Ethics Statement

The animal study was reviewed and approved by Administrative Panel on Laboratory Animal Care (APLAC) at Stanford University and the University of Pittsburgh.

## Author Contributions

WF, LC, and GL did the experiments and analyzed the data. GL drafted the manuscript. WF, LC, and SW edited the manuscript. SW and GL acquired funding and administrated the project. PN interpreted the aorta data from Tabula Muris project and edited the manuscript.

### Conflict of Interest

The authors declare that the research was conducted in the absence of any commercial or financial relationships that could be construed as a potential conflict of interest.

## Supplementary Material

Figure S1 Gender analysis of the ECs profiled by *Tabula Muris* in multiple organs **(A)**, heart chambers **(B)**, or aorta **(C)**.

Figure S2 The UMAP plots of Fam167b expression in aorta ECs profiled by *Tabula Muris* **(A)** and Lukowski et al. (22) **(B)**.

Figure S3 The merged PLISH staining images with a high resolution.

Table S1 The organ-specific EC genes. Function FindMarkers in Seurat 2 was used to identify the genes, and default parameter Wilcox test was used to calculate the *p* values.

Table S2 Cluster 9 specific genes. Seurat V2 and the default parameters were applied in this analysis.

Table S3 The genes specifically expresses in each germ layer derived ECs. Seurat V2 and default parameters were used for this analysis.

Table S4 The genes that differentially expressed in each heart EC populations. Seurat V2 and default parameters were used.

Table S5 The conserved genes that differentially expressed in each aorta EC population across three studies. Function FindMarkers in Seurat V3 with the default Roc test was used.

Table S6 The sequence of H probes used in PLISH experiments.
