# Supplementary figures and images for: Single Cell Analysis of Endothelial Cells Identified Organ-Specific Molecular Signatures and Heart-Specific Cell Populations and Molecular Features
Source: Front Cardiovasc Med. 2019 Nov 26;6:165. doi: 10.3389/fcvm.2019.00165 (PMC6901932; doi:10.3389/fcvm.2019.00165)

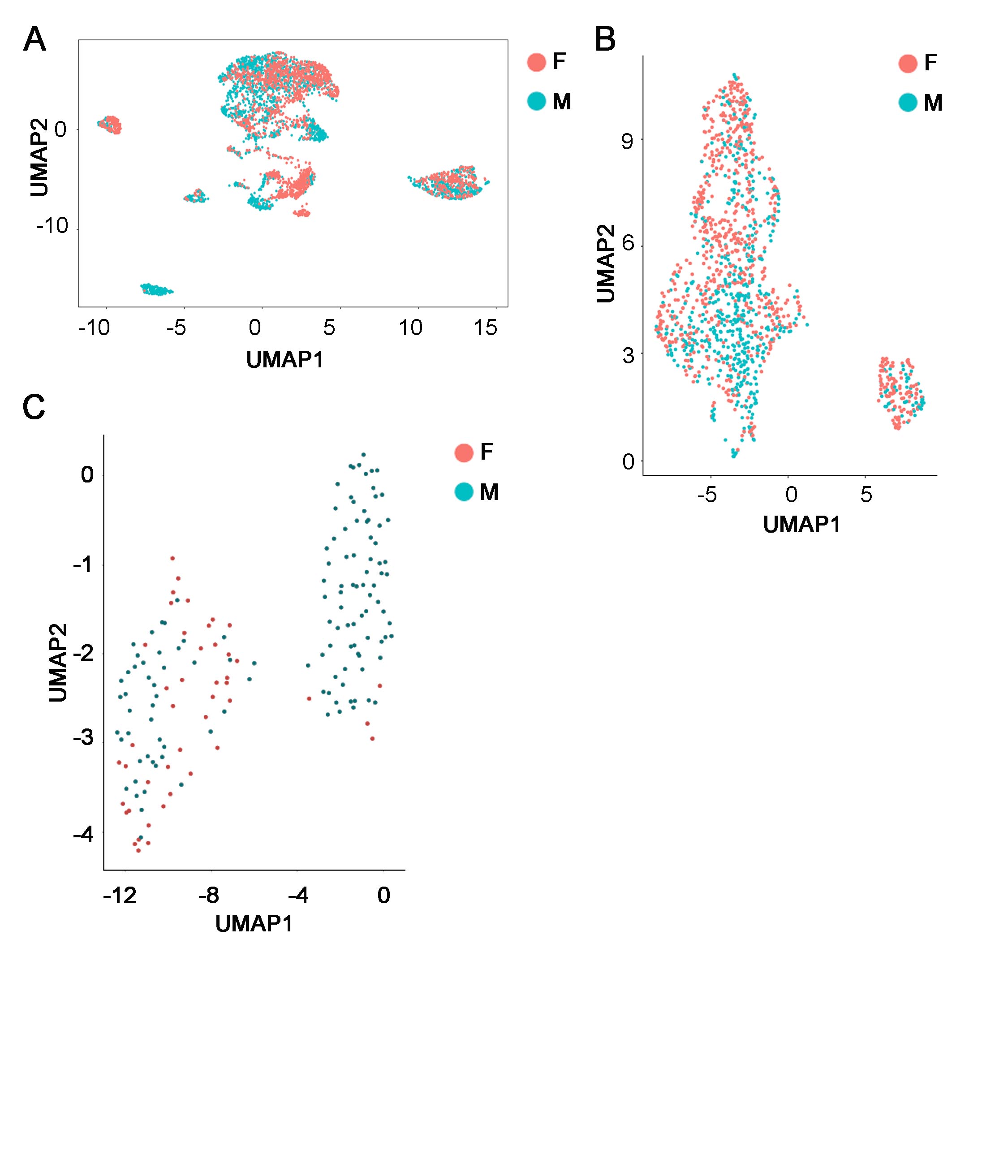

Supplement: Figure S1 — Gender analysis of the ECs profiled by Tabula Muris in multiple organs (A), heart chambers (B), or aorta (C). [file Image_1.JPEG]

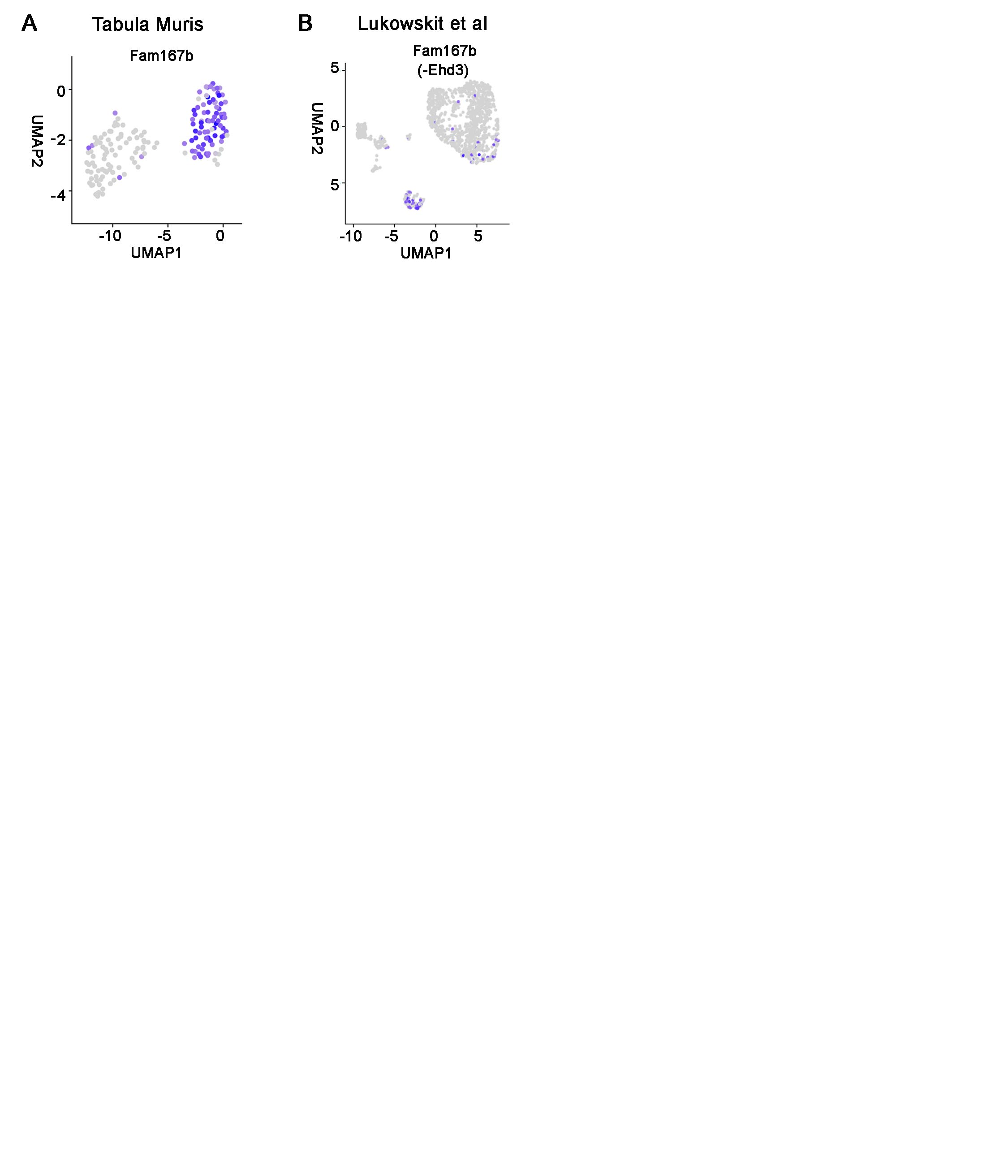

Supplement: Figure S2 — The UMAP plots of Fam167b expression in aorta ECs profiled by Tabula Muris (A) and Lukowski et al. (22) (B). [file Image_2.JPEG]

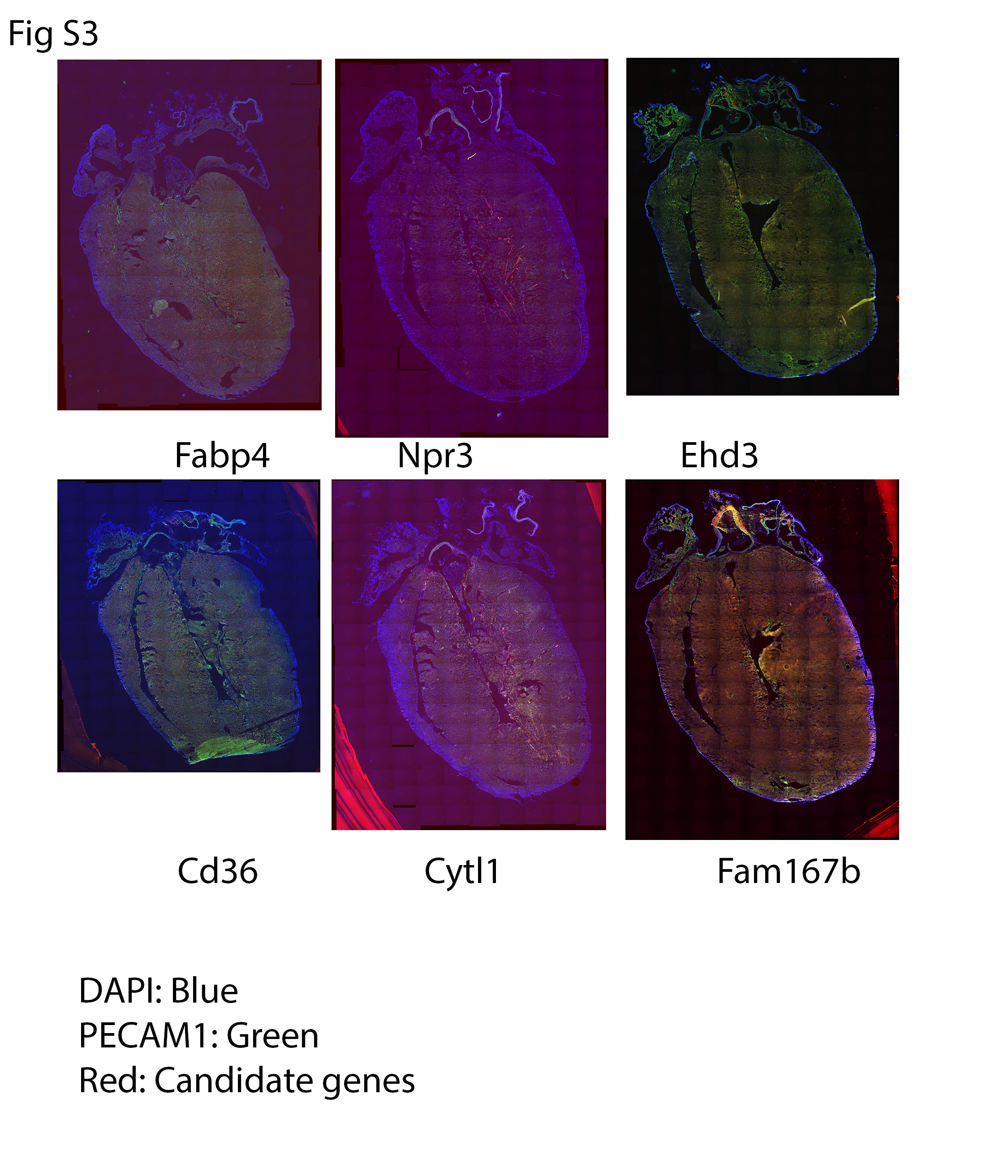

Supplement: Figure S3 — The merged PLISH staining images with a high resolution. [file Image_3.JPEG]
